# Impact of the Types and Relative Quantities of IGHV Gene Mutations in Predicting Prognosis of Patients With Chronic Lymphocytic Leukemia

**DOI:** 10.3389/fonc.2022.897280

**Published:** 2022-07-12

**Authors:** Matthew Kaufman, Xiao-Jie Yan, Wentian Li, Emanuela M. Ghia, Anton W. Langerak, Laura Z. Rassenti, Chrysoula Belessi, Neil E. Kay, Frederic Davi, John C. Byrd, Sarka Pospisilova, Jennifer R. Brown, Mark Catherwood, Zadie Davis, David Oscier, Marco Montillo, Livio Trentin, Richard Rosenquist, Paolo Ghia, Jacqueline C. Barrientos, Jonathan E. Kolitz, Steven L. Allen, Kanti R. Rai, Kostas Stamatopoulos, Thomas J. Kipps, Donna Neuberg, Nicholas Chiorazzi

**Affiliations:** ^1^Karches Center for Oncology Research, The Feinstein Institutes for Medical Research, Northwell Health, Manhasset, NY, United States; ^2^The Robert S. Boas Center for Genomics & Human Genetics, The Feinstein Institutes for Medical Research, Northwell Health, Manhasset, NY, United States; ^3^Center for Novel Therapeutics, Moores Cancer Center, University of California, San Diego, La Jolla, CA, United States; ^4^Laboratory Medical Immunology, Department of Immunology, Erasmus MC, University Medical Center, Rotterdam, Netherlands; ^5^Hematology Department, Nikea General Hospital, Pireaus, Greece; ^6^Division of Hematology, Mayo Clinic, Rochester, MN, United States; ^7^Department of Biological Hematology, Hôpital Pitié-Salpêtrière (AP-HP), Sorbonne Université, Paris, France; ^8^Department of Internal Medicine, University of Cincinnati College of Medicine, Cincinnati, OH, United States; ^9^Department of Internal Medicine - Hematology and Oncology and Department of Medical Genetics and Genomics, University Hospital Brno and Faculty of Medicine, Masaryk University, Brno, Czechia; ^10^Chronic Lymphocytic Leukemia Center, Dana-Farber Cancer Institute, Boston, MA, United States; ^11^Clinical Hematology, Belfast City Hospital, Belfast, Ireland; ^12^Department of Molecular Pathology, Royal Bournemouth Hospital, Bournemouth, United Kingdom; ^13^Department of Hematology, Royal Bournemouth Hospital, Bournemouth, United Kingdom; ^14^Department of Hematology & Oncology, Niguarda Cancer Center, Niguarda Hospital, Milan, Italy; ^15^Hematology Unit, Department of Medicine-(DIMED), University of Padua University Hospital, Padua, Italy; ^16^Department of Molecular Medicine and Surgery, Karolinska Institutet, Stockholm, Sweden; ^17^Division of Experimental Oncology, IRCCS Ospedale San Raffaele, Milan, Italy; ^18^Department of Molecular Medicine, Donald and Barbara Zucker School of Medicine at Hofstra/Northwell, Uniondale, NY, United States; ^19^Departments of Medicine, Donald and Barbara Zucker School of Medicine at Hofstra/Northwell, Uniondale, NY, United States; ^20^Northwell Health Cancer Institute, Lake Success, NY, United States; ^21^Institute of Applied Biosciences, Centre for Research and Technology Hellas, Thessaloniki, Greece; ^22^Department of Data Science, Dana-Farber Cancer Institute, Boston, MA, United States

**Keywords:** chronic lymphocytic leukemia, CLL, somatic mutations, immunoglobulin variable domain, prognosis

## Abstract

Patients with CLL with mutated IGHV genes (M-CLL) have better outcomes than patients with unmutated IGHVs (U-CLL). Since U-CLL usually express immunoglobulins (IGs) that are more autoreactive and more effectively transduce signals to leukemic B cells, B-cell receptor (BCR) signaling is likely at the heart of the worse outcomes of CLL cases without/few IGHV mutations. A corollary of this conclusion is that M-CLL follow less aggressive clinical courses because somatic IGHV mutations have altered BCR structures and no longer bind stimulatory (auto)antigens and so cannot deliver trophic signals to leukemic B cells. However, the latter assumption has not been confirmed in a large patient cohort. We tried to address the latter by measuring the relative numbers of replacement (R) mutations that lead to non-conservative amino acid changes (Rnc) to the combined numbers of conservative (Rc) and silent (S) amino acid R mutations that likely do not or cannot change amino acids, “(S+Rc) to Rnc IGHV mutation ratio”. When comparing time-to-first-treatment (TTFT) of patients with (S+Rc)/Rnc ≤ 1 and >1, TTFTs were similar, even after matching groups for equal numbers of samples and identical numbers of mutations per sample. Thus, BCR structural change might not be the main reason for better outcomes for M-CLL. Since the total number of IGHV mutations associated better with longer TTFT, better clinical courses appear due to the biologic state of a B cell having undergone many stimulatory events leading to IGHV mutations. Analyses of larger patient cohorts will be needed to definitively answer this question.

## Introduction

Patients with chronic lymphocytic leukemia (CLL) whose leukemic clone uses a mutated immunoglobulin heavy variable (IGHV) gene (M-CLL) typically have less aggressive disease than patients with CLL that use an unmutated IGHV gene (U-CLL) (1, 2). This observation has had a major direct impact on predicting the prognosis of CLL and a significant influence on its understanding and management (3, 4). Documenting this gene distinction is now considered a reliable prognostic factor, and the International Workshop on CLL (5) recommend checking this as a guideline for patient care and management. Moreover, IGHV-mutation status can help predict outcome for patients treated with chemoimmunotherapy (fludarabine, cyclophosphamide, and rituximab) (6–8), and for U-CLL patients treated with ibrutinib vs. chemoimmunotherapy (9). Moreover, IGHV-mutation status, along with other parameters, is incorporated into several prognostic algorithms (10–12).

There is speculation that the relationship between less aggressive disease and expression of mutated IGHVs is due to a loss or attenuation of autoreactivity of membrane immunoglobulin (IG), a major component of the B-cell antigen receptor (BCR), which limits the ability of the receptor to deliver trophic signals to the leukemic B cells. There is ample support for this concept. For example, U-CLL-derived IGs are extensively autoreactive, binding multiple self-molecules (13–15), especially those generated by apoptosis and protein catalysis (16–18). These are often referred to as natural autoantibodies. In contrast, M-CLL IGs are much less autoreactive. Notably, reverting M-CLL IGs to their germline sequence can lead to autoantigen binding, implying that those B cells that became leukemic *in vivo* might have been self-reactive prior to accumulating IGHV mutations (17, 19, 20). Thus, the process of losing autoantigen binding by somatic IGHV mutations can occur normally during B cell maturation, validating the speculation that this could explain the extended clinical course of patients with M-CLL. Additionally, CLL clones differ in their responsiveness to BCR engagement, with surrogate antigen binding, e.g., interaction with anti-IG antibodies, being more effective in stimulating U-CLL than M-CLL cases (21–23), and the ability to deliver a signal *via* the BCR correlating with worse clinical outcomes (24, 25). Finally, and possibly most convincingly, inhibition of BCR signaling by blocking the action of Bruton’s tyrosine kinase (BTK) (26–29) or of phosphoinositide 3’ kinase delta (PI3Kδ) (30–33) has a very significant effect on CLL cell survival, growth, and trafficking (9, 34–38). Such signaling inhibitors have had a major impact on the quality of patient lives, along with very high overall response rates and, in combination with other reagents, improving overall survival (39–42).

Nevertheless, the concept that the loss of polyreactive antigen binding and BCR signaling is at the root of better prognosis has not been directly confirmed in a large patient cohort, and that correlation is the intention of this investigation and report. For this process to be in play in most instances, only replacement (R) mutations and, in particular non-conservative R (Rnc) mutations, would be most relevant, since only R, and especially Rnc mutations can change the amino acid composition of an IGHV-IGHD-IGHJ rearrangement, thereby potentially altering (auto)antigen binding and eliminating or reducing BCR signaling. Conservative R (Rc) amino acid changes are less likely to alter protein structure and thereby (auto)antigen binding, and silent (S) mutations, by definition, cannot. Hence, since Rnc mutations more often alter amino acid structure, they are more prone to reduce BCR binding, and preempt cell signaling. In general terms, Rnc mutations yield an amino acid that has features opposite or distinct from those of the original one, e.g., hydrophilic vs. hydrophobic or non-polar vs. charged polar amino acids (43, 44). Additionally, in some instances, only a single R can lead to major alterations in protein structure and result in disease, e.g., cystic fibrosis and sickle cell anemia.

Here, we investigated the roles of S, Rc, and Rnc mutations on time-to-first-treatment (TTFT) of patients with CLL. This was addressed using a database of IGHV-IGHD-IGHJ DNA sequences with linked clinical information obtained from institutions in the United States of America and Europe. Our findings suggest that the relative frequencies of (S + Rc) IGHV mutations, which are less likely to create a major BCR structural change, are as important and correlate equally well with improved clinical course as Rnc mutations that are more likely to create a major BCR structural change. Moreover, our data suggest that the total number of mutations in the clonotypic rearranged IGHV gene of a CLL cell might be more central to better prognosis, suggesting that an overriding reason that IGHV mutations associate with better clinical course is the biologic state of a B cell that has undergone several rounds of stimulation leading to germinal center reactions, possibly varying in the follicular and extra-follicular types.

## Methods

### Patient Information and IGHV Sequence Data

Patient and corresponding IGHV-IGHD-IGHJ DNA sequence information (n = 3,598) were received from two large consortia studying CLL and BCR structure: the NIH-sponsored CLL Research Consortium (CRC) and the European Research Initiative on CLL/ImMunoGeneTics (ERIC/IMGT). CLL was defined as suggested by the latest guidelines from the International Workshops on CLL (5).

### Analysis of Immunogenetic Characteristics

The same IGMT software and tools were used by both consortia to analyze IGHV-IGHD-IGHJ sequences from the leukemic clones of CLL patients and to characterize and define if IGHV mutations could change amino acid sequence (S vs. R) and if R mutations were conservative (Rc) or non-conservative (Rnc), as determined by charge, hydropathy, and size.

### Analysis of Time to First Treatment

TTFT was defined as the number of years between the date of diagnosis and the date of initial therapy. The *survival* package of the R (statistical computing platform: https://www.r-project.org/) was used to estimate TTFT (Kaplan-Meier plots) through the *survfit* function. The impact of an independent variable on TTFT was determined by the Cox proportional hazard model (Cox regression) using the *coxph* function. To create graphic representations of TTFT in the figures in this document, Prism software and the log rank test were used. Nominal *P*-values are presented, without adjustment for multiplicity of testing.

### Division of Individual IGHV-IGHD-IGHJ Sequences Into Groups Based on the Ratio of IGHV Mutations More or Less Prone to Lead to a Significant BCR Structural Change

IGHV-IGHD-IGHJ gene rearrangement sequences were segregated based on the ratio of the combined number of S + Rc IGHV mutations divided by the number of Rnc IGHV mutations: (S+Rc)/Rnc. To allow mathematical comparisons should any type of mutation be absent, a value of 0.05 was added to each ratio component, (S+Rc +0.05)/(Rnc+0.05). For simplicity, the latter is represented in the text as “(S+Rc/Rnc”. An arbitrary (S+Rc)/Rnc percentage cutoff was chosen so that the calculated value would indicate the likelihood that amino acid change could appreciably alter BCR structure. Sequences with values of ≤ 1.0, based on the (S+Rc)/Rnc calculation, were considered more likely to lead to a major BCR structural change and are referred to as being in the “Low Ratio Group”; those sequences with values of > 1.0, were considered less likely to lead to a major structural change and are referred to as being in the “High Ratio Group”. Also, when another threshold cutoff value was used, i.e., the median value of all (S+Rc)/Rnc percentages (1.91), the reported findings were essentially the unchanged (**Figure S1**). For sequences with no IGHV somatic mutation, (S+Rc)/Rnc is not defined.

### Multiparameter Analysis of TTFT Comparing (S+Rc)/Rnc Percentage and Total Number of Mutations, Regardless of Type (S, Rc, Rnc)

A two-variable Cox regression was used to compare the total number of mutations (≥ 1) as the first variable, and logarithm-transformed mutation type ratio (S+Rc+0.5)/(Rnc+0.5) as the second variable, was used. A log-transformed ratio variable was employed as that more accurately followed a normal distribution than the ratio variable itself. The underlying assumption of the multivariable Cox regression was that the total number of mutations and the log-ratio jointly contribute to TTFT in an additive manner, and the contribution is averaged over the entire range of the variable values.

## Results

### Comparing and Coalescing the IGHV-IGHD-IGHJ Gene Rearrangement Sequence Data From the Two Consortia

To assure that merging the IGHV-IGHD-IGHJ gene rearrangement data from the CRC and ERIC/IMGT was appropriate, the distribution of sequences bearing the various types of somatic mutations was compared (**Table 1**). The sequence data from the CRC were collected from 1,690 patients with CLL; 36% were 100% unmutated (0 Mut), 2% had only S mutations (S only), 7% had only R mutations (R only), and 55% had a combination of S and R mutations (S+R). The DNA sequences from ERIC/IMGT were from 1,908 patients; 34% were 0 Mut, 1.6% S only, 6.4% R only, and 58% S+R. Thus, the IGHV mutations were similar in types, patterns, and distributions between the two data sets.

**Table 1:** Distributions of mutation types between the CLL Research Consortium (CRC) and the European Research Initiative on CLL/ImMunoGeneTics (ERIC/IMGT).

	CRC	ERIC/IMGT	Total
**0 mutations** **(100% homology with germline)**	36.0% (609)	34.1% (650)	35.0% (1,259)
**Silent (S) mutations only**	2.0% (34)	1.6% (31)	1.8% (65)
**Replacement (R) mutations only**	6.8% (114)	6.4% (122)	6.6% (236)
**Silent + Replacement mutations (S+R)**	55.2% (933)	57.9% (1,105)	56.6% (2,038)
**Total**	100% (1,690)	100% (1,908)	100% (3,598)

Moreover, although the methodologies to obtain IGHV-IGHD-IGHJ sequence data were not uniform among all the institutions in the two consortia, the same IMGT tools were used to analyze the data.

Therefore, based on very similar patient mutation parameters and uniform analytic approaches, the data from the two sites were pooled and used in the findings described here. The breakdown for the combined IGHV sequence data for the combined 3,598 patients with CLL was 35% 0 Mut (n = 1,259), 1.8% S only (n = 65), 7% R only (n = 236), and 57% S+R (n = 2,038).

### TTFT Defined by the Classical IGHV-Mutation Status Approach

**Figure 1:**
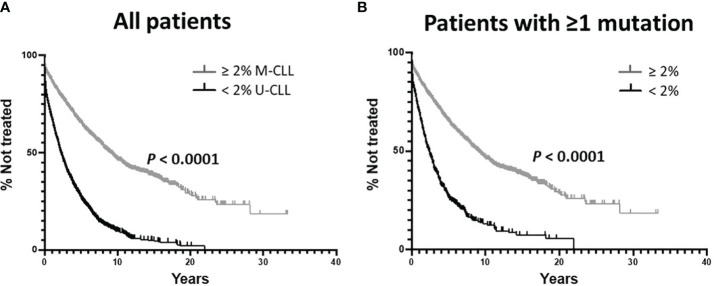
Kaplan-Meier estimates of time to first treatment (TTFT) using the classical IGHV-mutation status parameters. **(A)** Comparison of TTFT based on the < 2% vs. ≥ 2% difference from the germline IGHV sequence. All 3,598 sequences were used without regard for the types of somatic IGHV mutations. M-CLL: 1,885 patients, 856 treated; U-CLL: 1,713 patients, 1,369 treated. Data analyzed using the Log-rank (Mantel-Cox) test. **(B)** Comparison of TTFT based on the < 2% vs. ≥ 2% difference from the germline IGHV sequence analyzing only those patients with ≥ 1 IGHV mutation. < 2%: 454 patients, 351 treated; ≥ 2% 1,885 patients, 856 treated.

As per convention, we first divided all 3,598 patients, using the standard 2% difference from germline cutoff, into IGHV-unmutated (U-CLL; n = 1,713) and IGHV-mutated (M-CLL; n = 1,885) subgroups, and then used the Kaplan-Meier approach to estimate TTFT. In this way, as expected, M-CLL patients had a significantly longer TTFT than U-CLL patients (**Figure 1A**; median TTFT: 9.00 vs. 2.22yrs; *P* < 0.0001), consistent with established, published clinical observations and assuring that the combined cohort was a fair representation of the real-world patient base.

### Categorizing IGHV-IGHD-IGHJ Gene Rearrangement Sequences Containing Somatic IGHV Mutations Into Those With Mutation Types More or Less Likely to Change BCR Amino Acid Structure

The most direct way to address BCR structural change as being responsible for differences in TTFT would be to compare the clinical courses of CLL patients bearing somatically mutated IGHVs comprised of only S mutations to those patients whose IGHVs have only R mutations, or preferably, solely Rnc mutations. This would require having an extremely large database containing sequences with at least 5-6 somatic mutations of solely one type, since, depending on the individual IGHV gene expressed by a CLL clone, 5 or 6 mutations are needed to exceed the 2% mutation difference from germline and hence be tested in the standard IGHV-mutation analysis. This was not possible for our patient cohort, since none of the IGHV sequences exhibiting only S mutations reached the required ≥ 5 level, and only 44 sequences contained ≥ 5 R only mutations.

Therefore, we devised an approach that incorporated all patient sequences with IGHVs containing ≥ 1 mutation of any type (n = 2,339), and then segregated these based on the ratios of S + Rc mutations divided by Rnc mutations, (S+Rc)/Rnc. Since BCR ratios ≤ 1.0 (“Low Ratio Group”) would have a greater number of Rnc changes, such mutations would more likely lead to significant alterations in (auto)antigen binding. Likewise, BCRs with ratios > 1.0 (“High Ratio Group”) would be skewed to having a greater number of S and Rc mutations that would be less likely to change (auto)antigen binding (see **Methods** for details).

**Figure 2:**
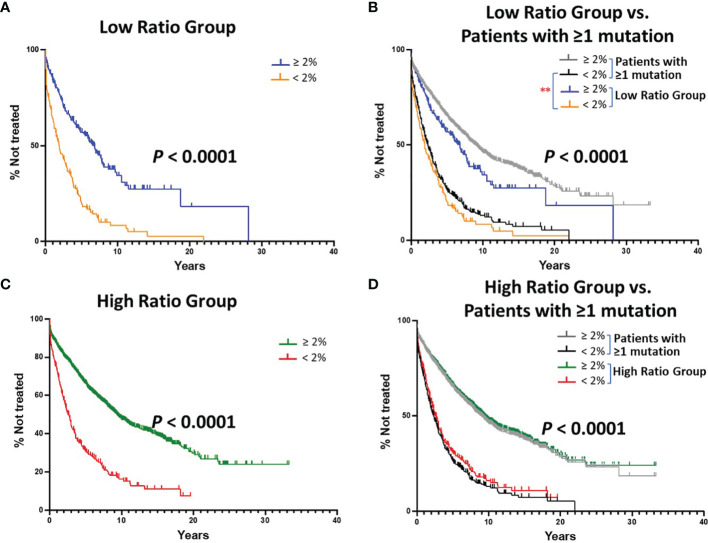
Kaplan-Meier estimates of TTFT of patients with IGHV sequences bearing at least 1 somatic mutation divided into Low or High (S+Rc)/Rnc Ratio Groups. **(A)** TTFT of patients with IGHV genes falling into the “Low Ratio Group”, ≤ 1.0 (S+Rc)/Rnc (n = 405) were compared based on the < 2% vs. ≥ 2% difference from the germline IGHV sequence. Number of cases in the < 2% difference group: 183, 152 treated (median TTFT = 1.91 years); number of cases in the ≥ 2% difference group: 222, 119 treated (median TTFT = 6.78 years) (P < 0.0001). **(B)** TTFT of all patients (**Figure 1B**) and those sequences in Low Ratio Group based on the < 2% vs. ≥ 2% difference from the germline IGHV sequence. ** = *P* < 0.01 Pair-wise Log-rank (Mantel-Cox) test. **(C)** TTFT of patients in the High Ratio Group, > 1.0 (S+Rc)/Rnc) (n = 1,934) compared based on the 2% cutoff. Number of cases in the < 2% difference group: 271, 199 treated (median TTFT = 2.59 years); number of cases in the ≥ 2% difference group: 1,663, 737 treated (median TTFT = 9.38 years) (P < 0.0001). **(D)** TTFT based on the < 2% vs. ≥ 2% difference from the germline IGHV sequence using all patient sequences (**Figure 1B**) and those sequences in High Ratio group. ** = *P* < 0.01.

After dividing the CLL patients with IGHV mutations in the original cohort into these two ratio groups, we went on to analyze TTFT. Thus, in the following analyses, the TTFT for patients in the Low and High Ratio Groups (**Figure 2**), as well as a subgroup that was created based on equal numbers of samples and mutations per sample (**Figure 5**), were compared in two ways: independently; after dividing each by the 2% IGHV-mutation cutoff; and based on the data obtained disregarding the < 2% or the ≥ 2% cutoff categories.

### TTFT for Patients in the Low and High Ratio Groups

When dividing the Low Ratio Group (n = 405) into categories below (worse outcome) and above (better outcome) the 2% cutoff, a clear and significant difference in TTFT was found (**Figure 2A**; median TTFT: 1.91 vs. 6.78 yrs; *P* < 0.0001). To illustrate how this result related to the standard IGHV-mutation analysis using all patient sequences (**Figure 1B**), the two sets of tracings were overlaid. This indicated that the > 2% category in the Low Ratio Group was different from the same category for all patients (**Figure 2B**; *P* < 0.01). Thus, the > 2% category for the Low Ratio Group has less patients in the better outcome category than the standard IGHV-mutation analysis using all patients; the statistical significance of this finding was not adjusted for multiple comparisons.

When segregating the High Ratio Group (n = 1,934) into the 2% cutoff categories and then comparing TTFT, again a highly significant difference was seen (**Figure 2C**; median TTFT: 2.59 vs. 9.38 years; *P* < 0.0001). Additionally, overlaying the curves from the High Ratio Group with those found using all IGHV-mutated patients (**Figure 1B**), TTFT for both the ≥ 2% and < 2% categories overlapped (**Figure 2C**). Thus, for the High Ratio Group, the relative numbers of patients in each of the two 2% cutoff categories are similar to those from the standard analysis using all patients (**Figure 2D**); this suggests that the High Ratio Group better reflects the IGHV mutation status distribution of the unseparated IGHV-mutated cohort.

**Figure 3:**
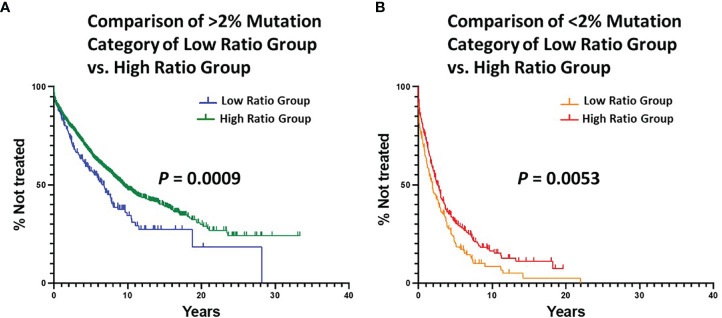
Comparison of estimated TTFT in the < 2% and ≥ 2% mutation categories of the Low Ratio Group and the High Ratio Group. **(A)** Comparison of TTFT in ≥ 2% mutation category of Low vs. High Ratio Groups (*P* = 0.0009). Number of cases from Low Ratio group: 222, 119 treated (median TTFT = 6.78 years). Number of cases from High Ratio group: 1663, 737 treated (median TTFT = 9.38 years). **(B)** Comparison of TTFT in < 2% mutation category of Low vs. High Ratio Groups (*P* = 0.0053). Number of cases from Low Ratio group: 183, 152 treated (median TTFT = 1.91 years). Number of cases from High Ratio group: 271, 199 treated (median TTFT = 2.59 years).

Next, we calculated the degree of difference in TTFT of the ≥ 2% and < 2% categories between the Low and High Ratio Groups. This indicated that the High Ratio Group had significantly longer TTFTs for the ≥ 2% (**Figure 3A**; median TTFT: 6.78 vs. 9.38 yrs; *P* = 0.0009) and the < 2% (**Figure 3B**; median TTFT: 1.91 vs. 2.59 yrs; *P* = 0.0053) categories than the Low Ratio Group.

**Figure 4:**
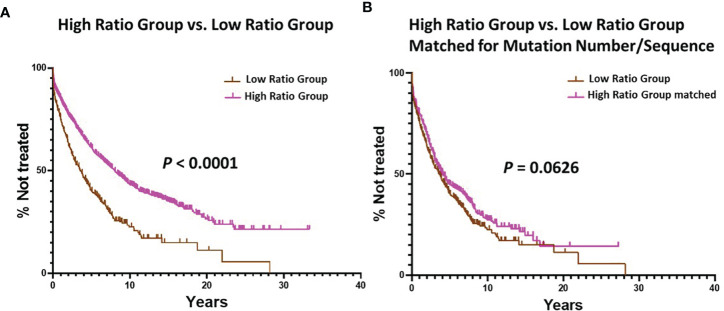
Comparison of estimated TTFT between the Low Ratio Group and the High Ratio Group. **(A)** Comparison of TTFT between the Low and High Ratio Groups using all patients in the respective groups (P < 0.0001). Number in Low Group = 405, median = 3.56 years; number in High Group = 1934, median = 8.03 years. **(B)** Comparison of TTFT after matching the Low and High Ratio Groups for equal numbers of patients (n = 405) with equal numbers of mutations per sequence (range: 1 – 36). Low Group median = 3.56 years, and High Group median = 4.08 years; *P* = 0.0626.

Finally, we compared TTFT for patients in the Low vs. High Ratio Groups without dividing them into < or ≥ 2% categories. This also indicated that the patients in the High Ratio Group had significantly better clinical courses than those in the Low Ratio Group (**Figure 4A**; median TTFT: 3.56 vs. 8.03 yrs; *P* < 0.0001).

Collectively, these calculations indicate that the both the High Ratio and the Low Ratio Groups contain patients with mutated IGHVs with better or worse clinical courses. Also, the inter-group comparisons suggest that the High Ratio Group might have a better TTFT than the Low Ratio Group in more accurately discerning important patient clinical outcomes.

### Correcting Analyses for Differences in the Numbers of Sequences of Various Types and in the Number of Mutations Per Type

The above estimates of TTFT using the various comparisons were unexpected in that they suggested that IGHV mutations which would likely change or likely not change BCR structure impact clinical course in a similar manner in general, and that the TTFT of High Ratio Group is even better. One confounding factor in the comparisons, however, is that the total number of patients in the Low Ratio Group is significantly less than in the High Ratio Group (Low: n = 405; High: n = 1,934). In addition, the DNA sequences of the individual patients in the High Ratio Group had a higher number of IGHV mutations (of any type) than the Low Ratio Group (**Figure S2**). Thus, a significantly smaller number of cases fell into the ≥ 2% category in the Low Ratio than the High Ratio Group (n = 222 vs 1,663).

Therefore, to rule out that the TTFT findings above were artefactual and not reflecting a true biologic effect, we modified the Groups to assure that the numbers of cases in the Low and High Ratio Groups were equal and that the numbers of mutations per sequence in each Group were similar. This was achieved using an exact match approach (45). Specifically, for each sample in the Low Ratio Group, a sample from the High Ratio Group was randomly picked that had the same number of total mutations. The end result was a one-to-one matching in sample size (n = 405) and mutation number between the Low and High Ratio Groups (range: 1 – 36).

**Figure 5:**
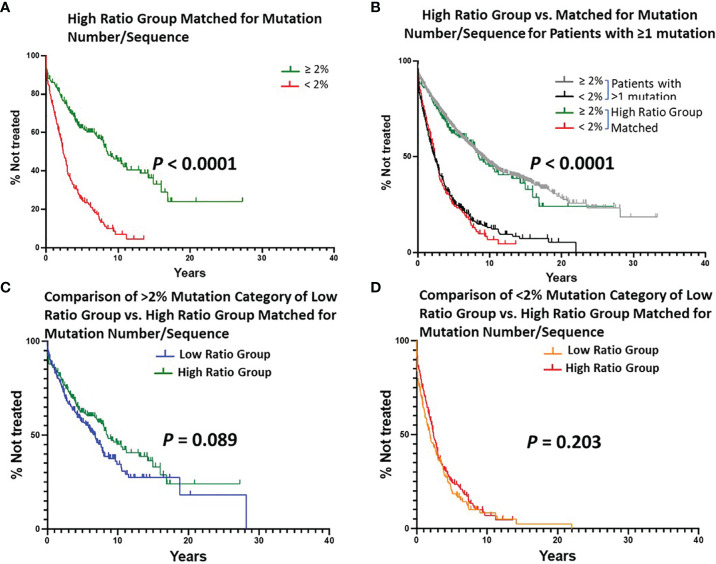
Estimated TTFT of patients in the Low or High Ratio Groups matched for numbers of patients and mutations per sequence. A, B, C, **(D)** Layout of graphs as per **Figure 2**. An exact matching approach with random sampling was used to achieve equal numbers of patients (n = 405) with the same number of IGHV mutations per patient (1-36) in the Low Ratio and High Ratio Groups. **(A)** Number of cases in the < 2% difference group: 187, 148 treated (median TTFT = 2.42 years); number of cases in the ≥ 2% difference group: 218, 101 treated (median TTFT = 8.50 years) (P < 0.0001). **(B)** TTFT based on the < 2% vs. ≥ 2% difference from the germline IGHV sequence using all patient sequences (**Figure 1B**) and those sequences in Matched High Ratio group. **(C)** Number of cases from Low Ratio group: 222, 119 treated (median TTFT = 6.78 years). Number of cases from Matched High Ratio group: 218, 101 treated (median TTFT = 8.50 years). **(D)** Number of cases from Low Ratio group: 183, 152 treated (median TTFT = 1.91 years). Number of cases from High Ratio group: 187, 148 treated (median TTFT = 2.42 years).

Since the number of cases in the Low Ratio Group was constant, the TTFT comparisons for this Group, based on the 2% cutoff, were those already shown in **Figure 2A** (*P* < 0.0001). The TTFT findings for the High Ratio Group were still very significant (**Figure 5A**; median TTFT: 2.42 vs. 8.50 yrs; *P* < 0.0001), and the curves based on the 2% cutoff overlapped that of the original High Ratio Group (**Figure 5B**). Moreover, this double matching approach reduced the differences between the Low and High Ratio Groups seen in **Figure 3** to insignificant levels (difference between > 2% categories: *P* = 0.089; difference between < 2% categories: *P* = 0.203).

Finally, we found that TTFT was equal for patients matched as above in the Low vs. High Ratio Groups without dividing them using the 2% cutoff (**Figure 4B**; TTFT: 3.56 vs. 4.08 yrs; *P* = 0.0626).

Collectively, these findings, especially when using cohorts of equal size and bearing the same number of mutations per sequence, suggest that the relative types of IGHV mutations with a higher (Low Ratio Group) or lower (High Ratio Group) probability of altering BCR structure have the same effect on TTFT. Thus, although somatic mutation altered BCR structure is likely a factor involved in lengthening TTFT in certain instances, it does not appear to be the most influential variable for this patient cohort.

### Total Numbers of Mutations, Regardless of Type, Correlate With TTFT After Exceeding a Minimum and Reaching an Apparent Maximum

**Figure 6:**
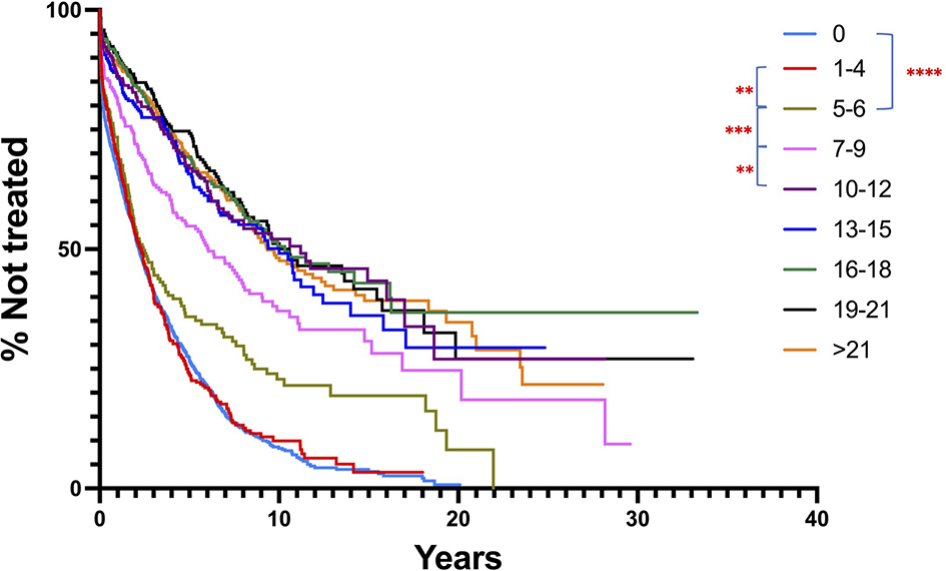
Comparison of TTFT for patients based on IGHV nucleotide mutation number intervals, regardless of mutation type. All patients with ≥ 1 mutation per IGHV sequence were segregated into nucleotide mutation number ranges, and then TTFT compared without using a 2% cutoff. Patients bearing clones without any IGHV mutations are provided for comparison. TTFT and numbers of patients in the various intervals: 1-4: median TTFT - 2.19 yrs, n = 389; 5-6: median TTFT - 2.43 yrs, n = 173; 7-9: median TTFT - 6.06 yrs, n = 232; 10-12: median TTFT - 11.21 yrs, n = 221; 13-15: median TTFT - 10 yrs, n = 257; 16-18: median TTFT - 10.33 yrs, n = 297; 19-21: median TTFT - 10.58 yrs, n = 240; >21: median TTFT - 9.36 yrs, n = 539. ** < 0.01; *** < 0.001.

Since both types of mutations appear to affect TTFT similarly, this implies that the total number of IGHV mutations might correlate better with clinical course in CLL. To test this, we re-ran the IGHV-mutation analysis on the original total patient cohort using all samples with ≥ 1 IGHV mutation, choosing a series of arbitrary mutation number cutoffs, ranging from 1 to > 21 (**Figure 6**). Notably, this approach indicated that the extent of TTFT increased significantly after the number of IGHV mutations reached and exceeded 5-6 mutations (the classical 2% cutoff) and reached a plateau for TTFT at ≥ 10 mutations. This again highlights that the ≥ 2% cutoff is effective in segregating cases into a better outcome group. Additionally, outcomes continue to improve as the number of total mutations increases at least to the level of ~10 mutations/sequence. Another study of patients treated with chemoimmunotherapy found that clinical outcome improved progressively as IGHV mutations increased (46). Notably, incorporating the (S+Rc)/Rnc variable into these arbitrary mutation intervals did not improve clinical course prediction (**Figure S3**).

### Multiparameter Analysis Comparing (S+Rc)/Rc Percentage and Total Number of Mutations, Regardless of Type, in Defining TTFT

Finally, we used a two-variable Cox regression to compare the total number of mutations (≥ 1) as the first variable, and logarithm-transformed mutation type ratio (S+Rc+0.5)/(Rnc+0.5) as the second variable (see **Methods**). The *P*-value for testing the null hypothesis that the total number of mutation variable does not contribute to the hazard ratio, is < 0.0001. The hazard ratio (increment of one mutation) is 0.955 (95% confidence interval: 0.948-0.962).

## Discussion

Defining the IGHV gene mutation status of a patient’s leukemic B cell clone is a cornerstone of prognosis in CLL (3, 47). Although several studies have addressed the best cutoff to be used in this analysis (46, 48–52), there has not been a detailed investigation aimed at determining if a loss of (auto)antigen – BCR interaction, which could obviate or reduce transmission of ongoing survival signals to the leukemic B cell, is the feature that is most responsible for defining M-CLL patients with better clinical outcomes. Here, we have tried to address this issue.

Although the most robust way to address the issue would be to compare the clinical courses and outcomes of CLL patients with IGHV genes expressing solely S vs. patients with solely R, especially Rnc, mutations, our database did not contain sufficient numbers of such cases with only S or only R or Rnc mutations to allow this. Therefore, we compared IGHV mutated sequences segregated based on skewing of the relative frequencies towards (S+Rc) or Rnc mutations, as the former are less likely to make a significant change in BCR structure than the latter. This was done for the entire population of samples with one or more somatic mutations and after normalizing the number of cases within the two (S+Rc)/Rnc High and Low Ratio Groups for numbers of samples and mutations per sequence.

Both relative mutation ratio options, < 1 and ≥ 1 (S+Rc)/Rnc, indicated similar TTFT, suggesting that the ability or inability to carry out BCR-mediated signaling is not the only reason that somatic mutations that lead to a ≥ 2% difference in nucleotide sequence influence clinical course.

However, several caveats need to be considered before excluding that a change in BCR structure is a major driver in determining clinical course. First, it is clear that for some antigens changing a single or only a very few amino acids in the IG variable domain can significantly alter auto- and foreign-antigen binding (53–55). This is the case for CLL B cells as well (56–58). Moreover, changing a single amino acid residue can affect the transmission of “autonomous BCR signaling” (59) based on BCR-BCR autoreactivity (60, 61), which appears to be a key factor in CLL B cell survival (59). Also, the apparent autoreactivity of IGs encoded by unmutated IGHV genes depends upon the proper pairing of IG heavy and light chains and the somatically generated VH CRD3s. As such, autoreactivity should not be considered an intrinsic property of unmutated rearranged IGHV genes commonly used in CLL (e.g., IGHV1-69), suggesting that autoreactivity can be a selected binding activity (19). Finally, it is possible that Rnc mutations lead to enhanced (auto)antigen binding and continuous binding site occupancy, thereby resulting in an anergic state (22, 62) that reduces cell division, clonal burden, and disease progression.

Second, our analyses only relate to potential structural changes involving the IGHV portion of the BCR. Changes in other parts, including the IGK/LV-IGK/LJ gene rearrangement and the VH CDR3 are not considered in this examination, and it is well documented that changes in each of these BCR regions are critical for (auto)antigen binding. Indeed, a characteristic mutation in the IGLV3-21 gene is directly associated with clinical course for CLL patients (63, 64), albeit in the opposite, more adverse, direction than would be predicted by IGHV-mutation analysis.

Nevertheless, the finding that TTFT increases as the number of total mutations increases is compatible with clinical courses being affected by the number of times a CLL cell precursor was signaled to undergo cell division and potentially a germinal center reaction. In this regard, the number of mutations accrued by a normal B lymphocyte is a function of the number of times a cell is stimulated to experience a germinal center reaction, with each episode leading to one mutation (65, 66). In this scenario, a given clinical course would not necessarily be directly affected by the types of IGHV mutations; rather, mutation amounts would imply the number of BCR signals received and the cell divisions undergone. This is consistent with the documentation that clinical outcomes of patients treated with chemoimmunotherapy are reflected by a continuum in the number of mutations a CLL B cells exhibits [**Figure 6** and (46)]. Thus, the physiologic state and the biologic properties of a cell that has undergone many rounds of stimulation and accumulated multiple mutations, regardless of a BCR structural change, could have a major prognostic impact. This situation is consistent with the phenotype (67), telomere (68), and methylation (69–71) features of CLL B cells, suggesting that all CLL cases, U-CLL as well as M-CLL, are derivatives of chronically stimulated, memory-like B cells. In this regard, it is important to recognize that there are several ways that antigen-experience and germinal center reactions can be initiated and where they can occur (72), e.g., within a lymphoid follicle in a classical germinal center with a well-defined cellular microenvironment, or outside of a follicle (extra-follicular), where the tissue architecture is not as rigidly set as in a germinal center within a follicle. Thus, the biologic characteristics of B cells that mutate in response to different types of (auto)antigenic challenges and within different tissue microenvironments might differ, as might the types and relative quantities of S, Rc, and Rnc mutations that are selected for or against. The similarities and differences in the follicular and extra-follicular B cell differentiation pathways are reviewed in (73, 74).

Finally, the two possibilities, i.e., BCR structural change and the number of CLL precursor B cell divisions, are not mutually exclusive. Certainly, the more often a cell undergoes a germinal center reaction the more likely a key R mutation could occur, especially since only a single or a few key amino acid changes can have dramatic influences on (auto)antigen binding. Moreover, the number of mutations in the IGHV gene, regardless of type, could be a surrogate for what has happened in the VH CDR3 or in the IGHK/LV-IGK/LJ genes. In this regard, one could postulate that the clinical courses of patients in the (S+Rc)/Rnc Low Ratio Group are a direct consequence of BCR structural changes in the IGHV domain of the BCR, and the clinical courses of those patients in the (S+Rc)/Rnc High Ratio Group could be the consequence of structural change that occurred outside of the IGHV domain. Future studies with larger numbers of CLL patient sequences might enable a more definitive understanding of the relative influences of these two parameters on the prognosis of CLL patients with the IGHV-mutated subtype.

## Data Availability Statement

Publicly available datasets were analyzed in this study. This data can be found here: https://www.imgt.org/CLLDBInterface/query.

## Ethics Statement

The studies involving human participants were reviewed and approved by Institutional Review Board, The Feinstein Institutes for Medical Research. The patients/participants provided their written informed consent to participate in this study.

## Author Contributions

MK and NC conceived the project. MK, X-JY, WL, DN, and NC designed the experimental and analytic approaches. MK, X-JY, WL, LR, and DN performed data analyses. X-JY, EG, AL, LR, CB, NK, FD, JoCB, SP, JRB, MC, ZD, DO, MM, LT, RR, PG, JaCB, JK, SA, KR, KS, TK, and NC provided clinical information and IGHV-IGHD-IGHJ DNA sequences. MK, X-JY, WL, EG, CB, NK, FD, ZD, DO, LT, RR, JoCB, JK, SA, TK, and NC edited the manuscript. All authors read the manuscript and agreed with the content.

## Funding

This study was supported in part by grants from the US National Institutes of Health, National Cancer Institute, PO1-CA81534 and RO1-CA236361 to TK and R01-CA238523 to NC. SP is supported by Ministry of Health of the Czech Republic, grant No. (NV19-03-00091). RR is supported by the Swedish Cancer Society, the Swedish Research Council, the Knut and Alice Wallenberg Foundation, Karolinska Institutet, Karolinska University Hospital, and Radiumhemmets Forskningsfonder, Stockholm. KS has received support from the Hellenic Precision Medicine Network in Oncology; and the project ODYSSEAS, funded by the Operational Programme “Competitiveness, Entrepreneurship and Innovation” (NSRF 2014–2020) and co-financed by Greece and the European Union, with grant agreement no: MIS 5002462. NC and KR thank the Karches Family, The Nash Family Foundation, and the Jean Walton Fund for Leukemia, Lymphoma, and Myeloma Research for their support of the Feinstein Institutes’ CLL Research & Treatment Program.

## Conflict of Interest

The following authors received funding from other sources as listed. However, these funders were not involved in the study design, collection, analysis, interpretation of data, the writing of this article or the decision to submit it for publication.

MK is Chief Executive Officer, StationMD; AL has received unrestricted research grants and/or speaker fees from Roche-Genentech, AbbVie, and Janssen. NK serves on Advisory Boards for AbbVie, Astra Zeneca, Beigene, Behring, Cytomx Therapy, Dava Oncology, Janssen, Juno Therapeutics, Onco tracker, Pharmacyclics and Targeted Oncology, is a member of Data Safety Monitoring Committees for Agios Pharm, AstraZeneca, BMS–Celgene, Cytomx Therapeutics, Janssen, Morpho-sys, Rigel, and received research funding from AbbVie, Acerta Pharma, Bristol Meyer Squib, Celgene, Genentech, MEI Pharma, Pharmacyclics, Sunesis, TG Therapeutics, Tolero Pharmaceuticals. JRB has served as a consultant for Abbvie, Acerta/Astra-Zeneca, BeiGene, Bristol-Myers Squibb/ Juno/Celgene, Catapult, Eli Lilly, Genentech/Roche, Hutchmed, Janssen, MEI Pharma, Morphosys AG, Novartis, Pfizer, Pharmacyclics, Rigel; received research funding from Beigene, Gilead, Loxo/Lilly, SecuraBio, Sun, TG Therapeutics. MM has received research funding from Roche-Genentech, has had advisory roles for Abbvie, Astra Zeneca, Gilead, Janssen Pharmaceuticals, and Verastem Oncology. RR has received honoraria from AbbVie, AstraZeneca, Janssen, Illumina, and Roche. PG has served on advisory boards or as a consultant for AbbVie, AstraZenenca, BeIgene, BMS, Janssen, Lilly/Loxo Merck, Roche Sanofy, and received research support from AbbVie, AstraZenenca, and Janssen. JoCB has received research support from Pharmacyclics/Abbvie and AstraZeneca, Oncternal, and Velosbio, and has served on advisory boards for Pharmacyclics/Abbvie, Beigene, AstraZeneca, TG Therapeutics, and MEI. SA owns stock in Bristol Myers Squibb and C4 Therapeutics and has served on the Advisory Boards of Stemline Therapeutics and Sanofi Genzyme. KS has received support from the Hellenic Precision Medicine Network in Oncology and the project ODYSSEAS funded by the Operational Programme “Competitiveness, Entrepreneurship and Innovation” (NSRF 2014–2020) and co financed by Greece and the European Union, with grant agreement no: MIS 5002462. TK has had consultancy/advisory roles for AbbVie, Genentech-Roche, Gilead, Pharmacyclics LLC, an AbbVie Company, and Celgene; and research funding from AbbVie, Genentech-Roche, Pharmacyclics LLC, an AbbVie Company, and Oncternal. DN has stock in Madrigal Pharmaceuticals and receives research funding from Pharmacyclics LLC, an AbbVie Company. NC has received research funding from Verastem Oncology, argenx, Janssen Pharmaceuticals, and AVA Lifesciences GmbH.

The remaining authors declare that the research was conducted in the absence of any commercial or financial relationships that could be construed as a potential conflict of interest.

## Publisher’s Note

All claims expressed in this article are solely those of the authors and do not necessarily represent those of their affiliated organizations, or those of the publisher, the editors and the reviewers. Any product that may be evaluated in this article, or claim that may be made by its manufacturer, is not guaranteed or endorsed by the publisher.
