# Supplementary material for: Impact of the Types and Relative Quantities of IGHV Gene Mutations in Predicting Prognosis of Patients With Chronic Lymphocytic Leukemia
Source: Front Oncol. 2022 Jul 12;12:897280. doi: 10.3389/fonc.2022.897280 (PMC9315922; doi:10.3389/fonc.2022.897280)
Supplement: Supplementary file 1 [file DataSheet_1.pdf]

Supplementary Data

Figure S1. **Comparison of estimated TTFT in the < 2% and ≥ 2% mutation categories of the Low Ratio Group and the High Ratio Group defined by median of all samples**. **A**. Comparison of TTFT of Low vs. High Ratio Groups (*P* = 0.0069). Number of cases from Low Ratio group:1188, 627 treated (median TTFT = 6.35 years). Number of cases from High Ratio group: 1151, 580 treated (median TTFT = 7.66 years); **B**. Comparison of TTFT in ≥ 2% mutation category of Low vs. High Ratio Groups (*P* = 0.0494). Number of cases from Low Ratio group: 954, 438 treated; median TTFT = 8.10 years. Number of cases from High Ratio group: 931, 418 treated; median TTFT = 9.58 years; **C**. Comparison of TTFT in < 2% mutation category of Low vs. High Ratio Groups (*P* = 0.0939). Number of cases from Low Ratio group: 235, 190 treated; median TTFT = 2.01 years. Number of cases from High Ratio group: 218, 161 treated; median TTFT = 2.49 years.

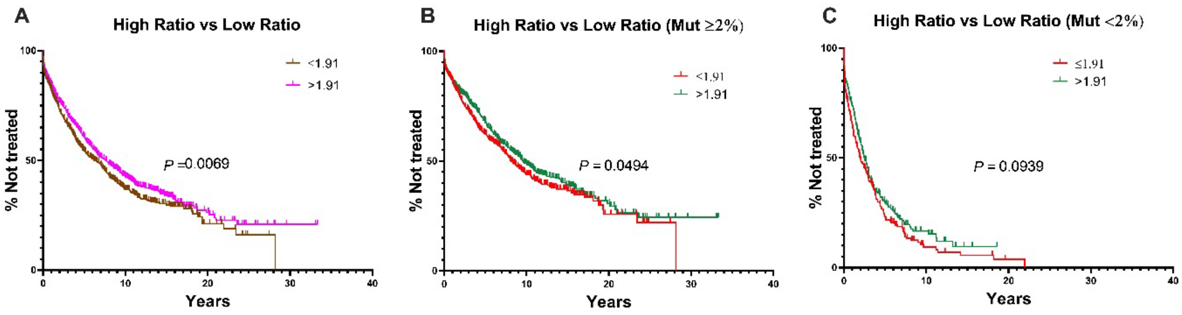

Figure S2. Scatter plot of (S+Rc)/Rnc ratio versus total number of mutations. Each point in the plot represents a patient who has at least one IGHV somatic mutation (n = 2,339). Noise was added in both x and y values in order to separate points with identical values. The (S+Rc)/Rnc ratio is defined by (S+Rc + 0.5)/(Rnc + 0.5), where a small fraction (0.5) is added in both numerator and denominator to avoid divergence. The y-axis is plotted in a logarithmic scale. The ratio of 1.0 and median ratio (1.9) are marked with red horizontal lines. When Rnc = 0, the ratio for (S+Rc) = 1,2,3, ... will be 3, 5, 7, ... and these points are highlighted in a blue line marked as Rnc = 0. Similarly, Rnc = 1, Rnc = 5, S+Rc = 0, S+Rc = 1, S+Rc = 5 points are also marked with blue lines.

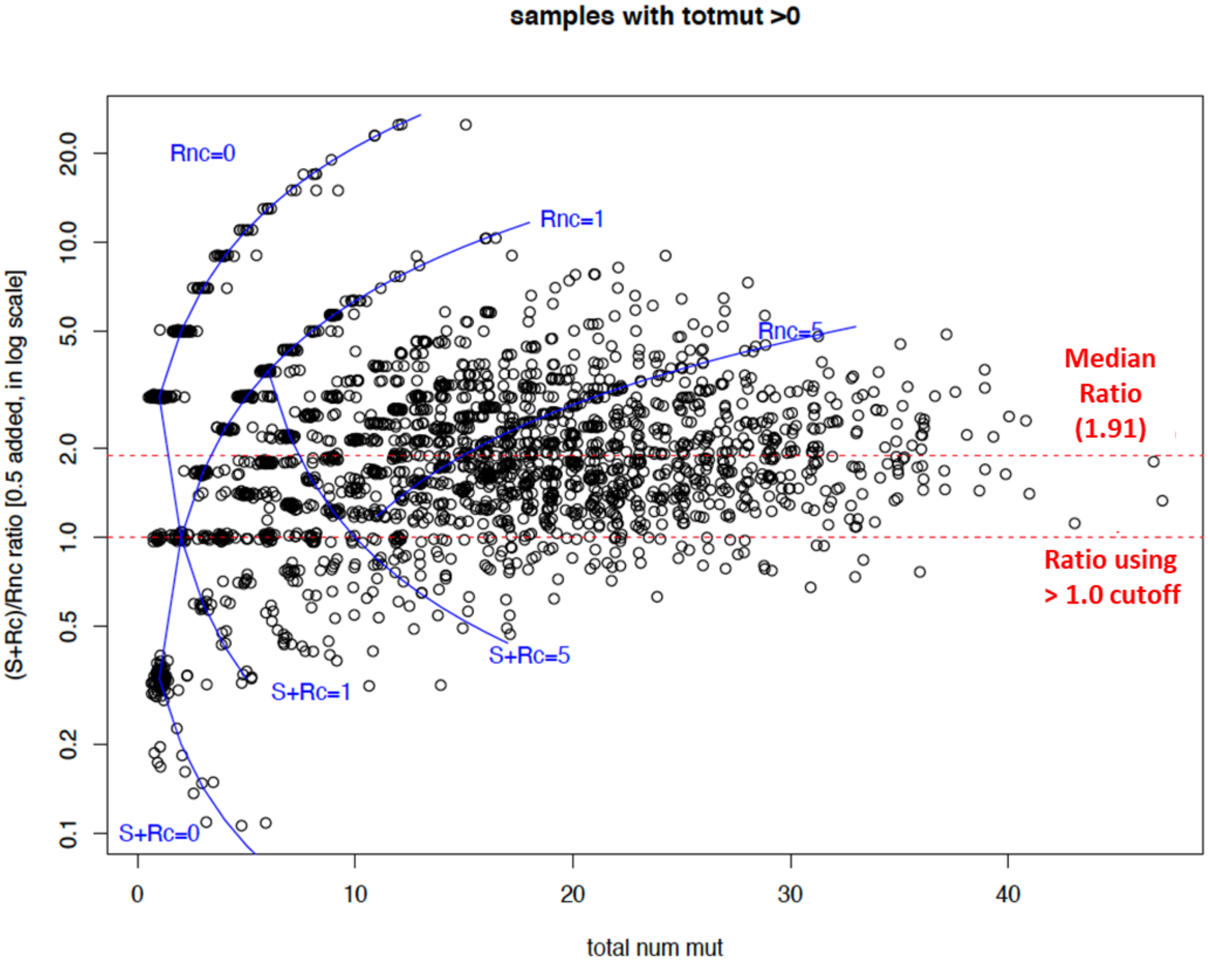

Figure S3. Influence of low (≤ 1) and high (> 1) (S+Rc) to Rnc ratios on estimated time-to-first-treatment (TTFT) of patients assigned to IGHV mutation intervals. Patients were assigned to a series of arbitrary IGHV mutation intervals (see **Figure 6**), and then divided into low (ratio ≤ 1) and high (ratio > 1) ratio groups based on (S+Rc)/Rnc.

A. Forest plot of hazard ratio (HR) and 95% confidence interval (CI) for (S+Rc)/Rnc ratio ≤ 1samples versus the ratio > 1 samples stratified by the number of total mutations. When the 95% CI crosses the HR = 1 value (indicated by a vertical line), the HR is not significantly different from the HR = 1 value at the 0.05 level.

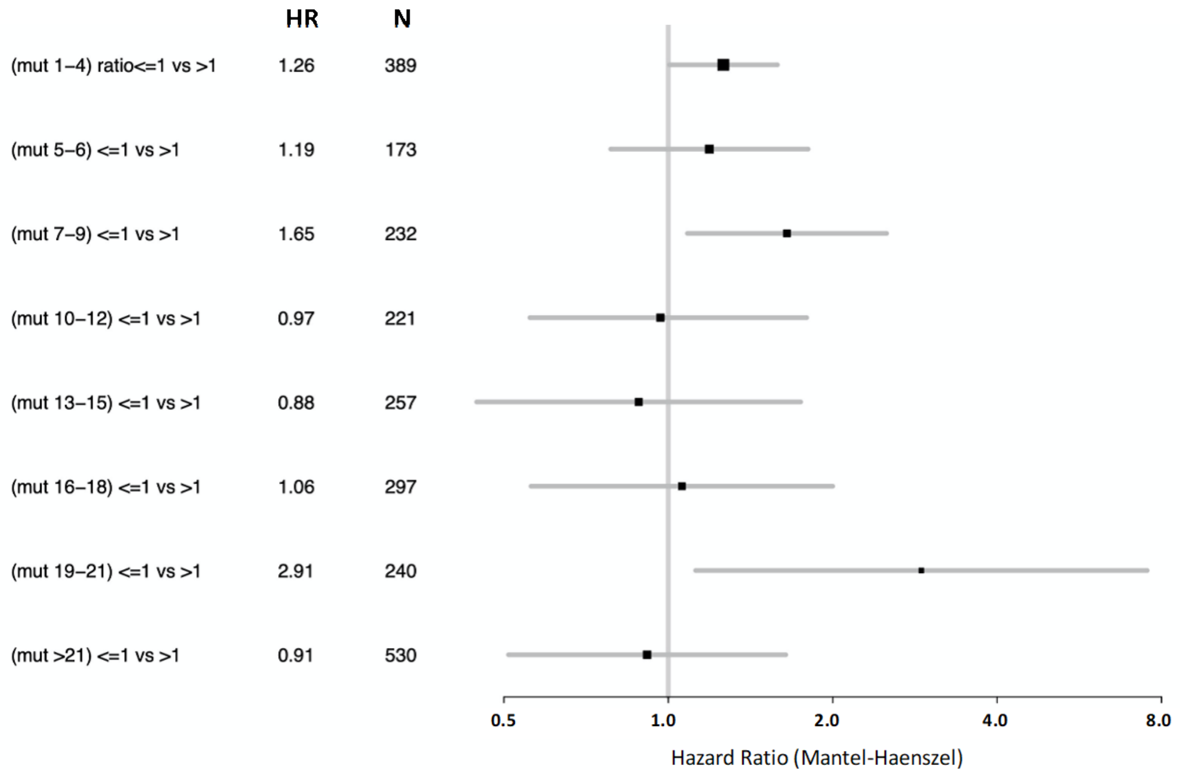

B. Figures illustrate the TTFT of the two ratio groups (ratio ≤ 1 and ratio > 1) for patients falling into the various IGHV mutation intervals. Table lists the numbers of patients in each group (total and treated), TTFT in years, and *P*-values for the various comparisons.

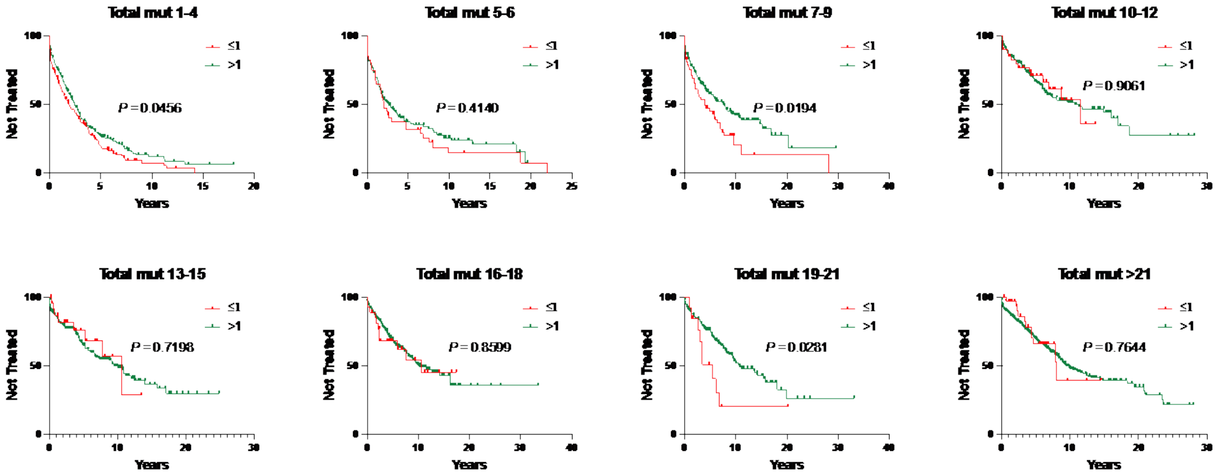

Total mutated Nt	0	1-4	5-6	7-9	10-12	13-15	16-18	19-21	>21	Total
# Patients	1259	389	173	232	221	257	297	240	530	
# Treated	1018	308	126	131	93	113	117	103	216	
Median TTFT (Years)	2.19	2.32	2.43	6.06	11.21	10.00	10.33	10.58	9.36	
Low Ratio Group (S+Rc)/Rnc ≤1	NA	173	40	53	40	24	27	13	35	405
		42.7%	9.9%	13.1%	9.9%	5.9%	6.7%	3.2%	8.6%	
Median TTFT (Years)		1.92	2.04	3.92	11.50	10.51	10.52	5.42	8.00	
High Ratio Group (S+Rc)/Rnc >1	NA	216	133	179	181	233	270	227	495	1934
		11.2%	6.9%	9.3%	9.4%	12.0%	14.0%	11.7%	25.6%	
Median TTFT (Years)		2.42	2.65	7.66	11.21	9.39	10.33	10.63	9.36	
P-value High vs Low		0.046	0.414	0.019	0.906	0.421	0.86	0.028	0.764	
